# Topological defects in the mesothelium suppress ovarian cancer cell clearance

**DOI:** 10.1063/5.0047523

**Published:** 2021-08-03

**Authors:** Jun Zhang, Ning Yang, Pamela K. Kreeger, Jacob Notbohm

**Affiliations:** 1 Department of Engineering Physics, University of Wisconsin-Madison, Madison, Wisconsin 53706, USA; 2 Biophysics Program, University of Wisconsin-Madison, Madison, Wisconsin 53706, USA; 3 Department of Biomedical Engineering, University of Wisconsin-Madison, Madison, Wisconsin 53706, USA; 4 Department of Cell and Regenerative Biology, University of Wisconsin School of Medicine and Public Health, Madison, Wisconsin 53705, USA; 5 Department of Obstetrics and Gynecology, University of Wisconsin School of Medicine and Public Health, Madison, Wisconsin 53705, USA; 6 University of Wisconsin Carbone Cancer Center, and University of Wisconsin School of Medicine and Public Health, Madison, Wisconsin 53792, USA

## Abstract

We investigated an *in vitro* model for mesothelial clearance, wherein ovarian cancer cells invade into a layer of mesothelial cells, resulting in mesothelial retraction combined with cancer cell disaggregation and spreading. Prior to the addition of tumor cells, the mesothelial cells had an elongated morphology, causing them to align with their neighbors into well-ordered domains. Flaws in this alignment, which occur at topological defects, have been associated with altered cell density, motion, and forces. Here, we identified topological defects in the mesothelial layer and showed how they affected local cell density by producing a net flow of cells inward or outward, depending on the defect type. At locations of net inward flow, mesothelial clearance was impeded. Hence, the collective behavior of the mesothelial cells, as governed by the topological defects, affected tumor cell clearance and spreading. Importantly, our findings were consistent across multiple ovarian cancer cell types, suggesting a new physical mechanism that could impact ovarian cancer metastasis.

## INTRODUCTION

Ovarian cancer has been shown to metastasize by hematogenous, lymphogenous, and transcoelomic spread. Of these modes, transcoelomic spread appears to be the dominant mechanism, as tumor cells metastasize by disconnecting from the primary tumor, floating in the peritoneal fluid, and re-attaching at new sites through adhesion to the mesothelium. Multiple mechanisms that regulate the adhesion step of this process have been identified, including interactions between tumor cell CD44 and mesothelial fibronectin,^1^ tumor cell β1 integrins and mesothelial extracellular matrix,^2,3^ and tumor cell CD24 and mesothelial P-selectin.^4^

To establish a niche within the new metastatic site, cancer cells subsequently invade into the mesothelial monolayer to access the underlying stroma in a process referred to as mesothelial clearance. Studies have identified biological mechanisms in tumor cells that promote this invasion, including the expression of mesenchymal transcription factors (*SNAI1*, *TWIST*, *ZEB1*),^5^ alcohol dehydrogenase 1B (*ADH1B*),^6^ and keratin-14 (*KRT14*).^7^ It has been shown that clearance of the mesothelial cell layer by ovarian cancer cells depends on the integrin-based interaction with the extracellular matrix^5,8–10^ and the actomyosin-based generation of force.^11^ Together, these observations implicate the importance of physics—namely, adhesion and force—in mesothelial clearance. However, most prior studies have focused on how variation between tumor cells affects the ability for clearance to occur; the role of the mesothelial layer in resisting this breach is less understood. For example, it is unknown how physical factors such as mesothelial cell orientation and motion within the monolayer impact clearance.

To investigate this question, we begin by considering how shape and motion are related in confluent cell layers. Motion within the cell layer is described by the vector field of velocity, and alignment between neighboring cells is described by the tensor field identifying the cell orientations. It is possible for the cell orientations to be discontinuous over space, which occurs at locations called topological defects. More precisely, if the cell orientations are defined by angle *θ* in the two-dimensional plane, then topological defects are defined as points for which *θ* is discontinuous. Such defects have been observed in monolayers of various cell types, including rod-shaped bacteria, eukaryotic cells with elongated fibroblast-like morphology, and eukaryotic cells with rounded epithelial morphology.^12–19^ Little is known about the existence of topological defects *in vivo*, though a recent study in *Hydra* has related defects in supracellular alignment of actin fibers to regeneration of the foot and head.^20^ In cell monolayers, topological defects can affect the pattern of cell motion, causing net outward or inward cell velocity, depending on the type of defect.^16,17,19,21^ In turn, the outward and inward velocities at the defects can produce holes or cause cells to extrude from the monolayer at the locations of the defects.^16,17,19^ Mesothelial cells may be subject to extrusion, as they are frequently identified in the cellular fraction of ascites in ovarian cancer patients.^22^ These findings raise the possibility that mesothelial cell orientation and velocity are related according to the theory and, further, that mesothelial clearance during cancer invasion may be altered by defects in the mesothelial cell layer.

In this study, we tested the hypothesis that clearance of mesothelial cells by ovarian cancer cells is altered by topological defects in the mesothelial cell layer. To begin, we first identified topological defects and quantified how local cell motion and density varied between regions with and without defects. We then used an *in vitro* model for mesothelial clearance in which spheroids of ovarian cancer cells were seeded on top of the mesothelial cell layer and quantified clearance in regions with or without topological defects.

## RESULTS

### Topological defects in mesothelial cell layers

**FIG. 1.**
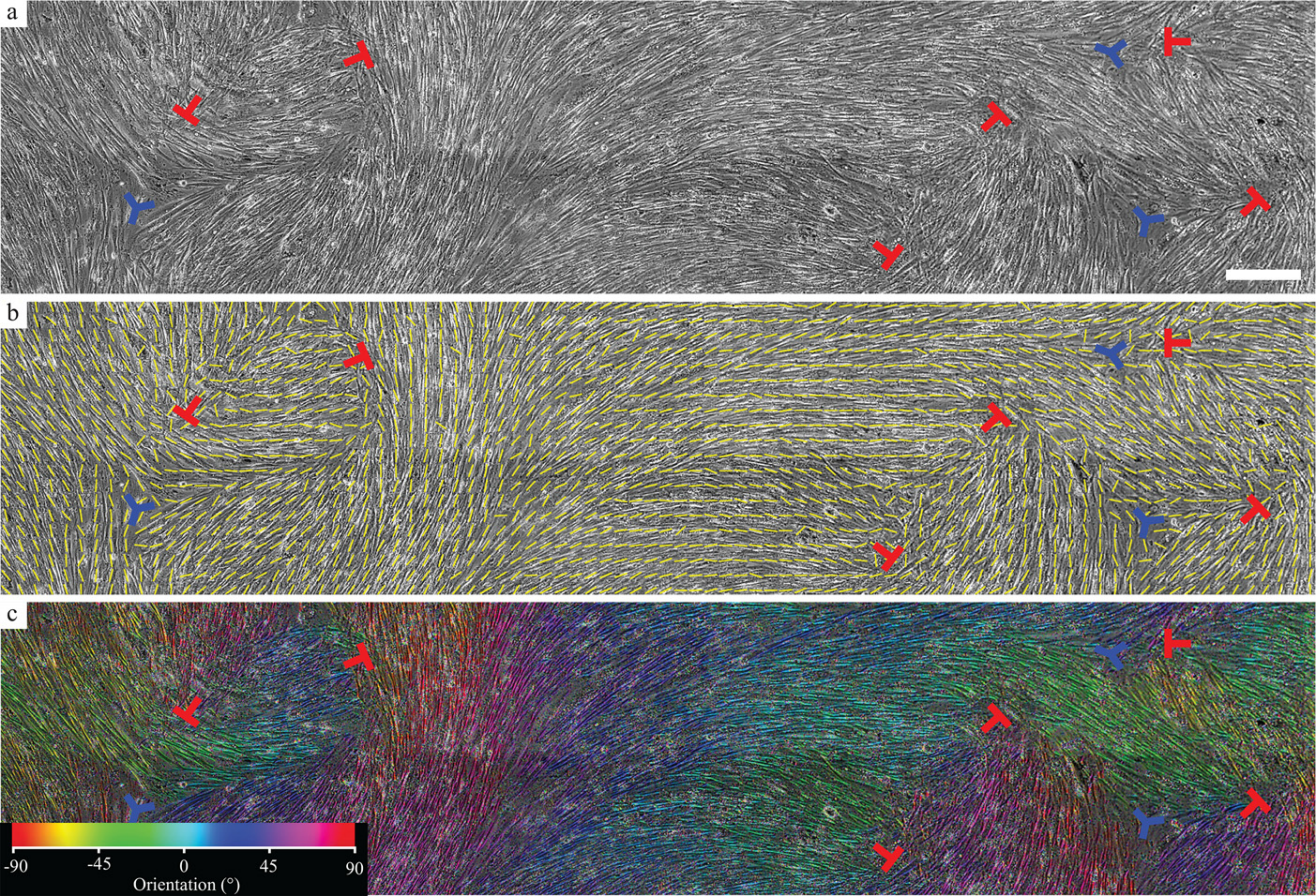
Topological defects in the mesothelial cell layer. (a) Representative phase contrast image of LP-9 mesothelial cells. (b) Same image as in panel (a) with the tensor field indicating cell orientations. (c) Same image as in panel (a) with colors indicating the angle of local cell orientation. Topological defects are indicated with red and blue symbols indicating +1/2 and −1/2 defects, respectively. Scale bar: 500 *μ*m.

We first analyzed the human mesothelial cell line LP-9 to determine if topological defects were present in confluent monolayers. These cells exhibited an elongated morphology with a high aspect ratio. To study alignment of LP-9 cells, a confluent layer of the cells was imaged [Fig. 1(a)], and the tensor field was mapped [Fig. 1(b)] enabling us to identify topological defects.^16^ One feature of these defects is that they separate domains of cells having different orientations [Fig. 1(c)]. At +1/2 defects, two domains are approximately perpendicular to each other. At −1/2 defects, three domains meet and are separated by angles of approximately 120°. The +1/2 defect has one axis of symmetry (the tail segment of the red ⊥, symbol in Fig. 1), which is sometimes referred to as a comet tail. The −1/2 defect has three axes of symmetry (blue segments in Fig. 1), which are hereafter referred to as three legs. Both types of defects were also observed in monolayers of primary human mesothelial cells isolated from benign omentum (Fig. 7). Full integer defects were not observed in our experiments.

### Cell velocities near topological defects

**FIG. 2.**
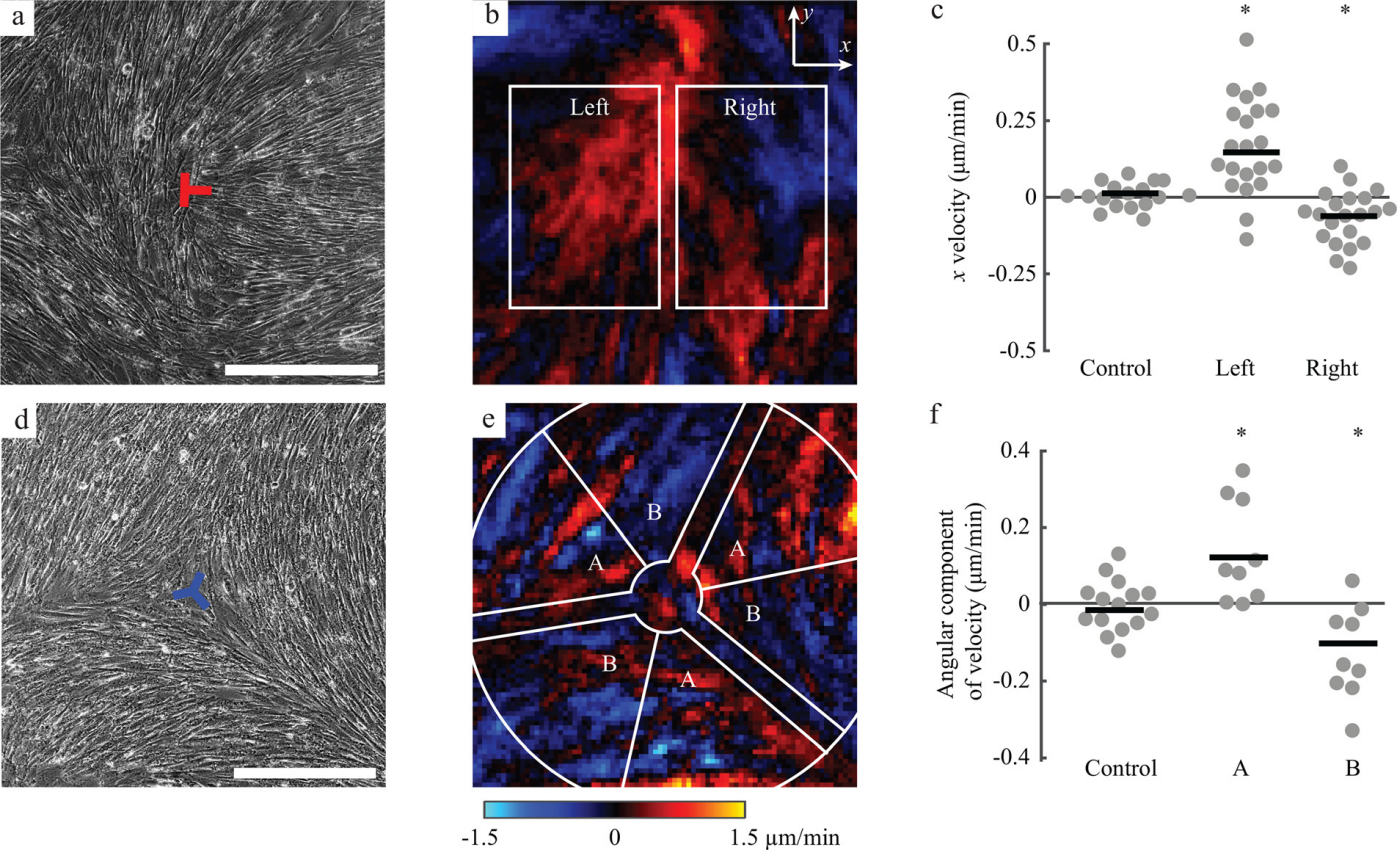
Collective mesothelial cell migration patterns near the defects. (a) Representative image of a +1/2 defect. (b) Colormap of the *x* component of cell velocity near the +1/2 defect shown in panel (a) averaged over 24 h. (c) Velocities were averaged within regions of width 500 *μ*m and height 750 *μ*m on the left and right side of the defect, as identified by the white boxes in panel (b). The plot shows the average *x* velocity of control positions having no defect (*n *=* *21) and in regions to the left (*p *<* *0.01) and right (*p *<* *0.05) of +1/2 defects (*n *=* *21). (d) Representative image of a −1/2 defect. (e) Colormap of the angular component of cell velocity near the −1/2 defect shown in panel (e) averaged over 24 h. (f) Velocities were averaged within the six regions on either side of the three legs of the −1/2 defect [labeled A and B in panel (e)]. Data points within a distance of 250 *μ*m from the center of the defect or within 45 *μ*m from the legs of the defect were excluded. The plot shows the average angular component of velocity of control positions (*n *=* *15) and of positions labeled A (*p *<* *0.01) and B (*p *<* *0.05) surrounding −1/2 defects (*n *=* *9 defects). Scale bars: 500 *μ*m.

Following observations of prior studies,^13–19^ we hypothesized that the +1/2 and −1/2 defects would alter patterns of cell motion. Therefore, we imaged multiple defects over time (Videos 1 and 2) and quantified cell velocities with digital image correlation. For +1/2 defects, we defined the *x* direction to be along the axis of the comet tail with the positive direction pointing toward the tail [Figs. 2(a) and 2(b)]. On the positive (right) side of +1/2 defects, the *x* component of cell velocity was negative with cells migrating toward the center of the defect [Figs. 2(b) and 2(c)]. Interestingly, on the left side of the defect, the *x* component of velocity was positive [Figs. 2(b) and 2(c)]. Thus, cells on both the sides of the defect moved inward. This inward motion was a common feature (observed in 16 out of 21 defects), resulting in a statistically different average *x* component of velocity compared to cell velocities at defect-free control regions in the cell layer [Figs. 2(c) and 8]. We considered that the velocity fields may have been altered by the fact that +1/2 defects move over time (whereas −1/2 defects do not).^15,23^ By reviewing the time lapse images (Video 1), it appeared that indeed +1/2 defects moved, but very slowly (∼1 *μ*m/h), resulting in an average total displacement of only 33 *μ*m over the course of an experiment (Fig. 9). As this displacement is smaller than the 50 *μ*m distance between the two regions used for analyzing the *x* component of velocity [Fig. 2(b)], the results were unaffected by motion of the defects. Velocities in the *y* direction near +1/2 defects were not statistically different from velocities in defect-free control regions in the cell layer [Figs. 10(a)–10(c)]. Considering that the results may have been affected by the large stiffness of the plastic dishes used for the experiments, we repeated the experiment on 3 kPa polyacrylamide gels, which match the stiffness of benign human omentum.^24^ On 3 kPa gels, cell velocity fields near +1/2 defects showed the same trend, namely, that cells moved inward toward the center of the defect (Fig. 11), suggesting that the substrate stiffness does not have a major effect on trends in cell velocities. In summary, the flow near +1/2 defects was along the *x* axis and toward the center of the defect.

To characterize cell velocities near −1/2 defects, we first computed the angular component of cell velocity [Figs. 2(d) and 2(e)]. Different positions around each −1/2 defect were classified into two groups (labeled as “A” and “B”) based on their local position with respect to the three legs of the −1/2 defect. Cells in regions labeled A migrated in the positive direction (counterclockwise), while those in B migrated in the negative direction [Fig. 2(f)]. These trends in the angular component of velocity were statistically different than the random cell velocity at defect-free control regions [Figs. 2(f) and 8(d)], indicating that the cells moved toward the three legs associated with each −1/2 defect. We also quantified the radial component of velocity near the −1/2 defects. On the legs, no systematic inward or outward migration was present, but outside of the legs, there was a net outward cell velocity that was statistically different from defect-free control regions [Fig. 10(f)].

### Local cell densities near topological defects

**FIG. 3.**
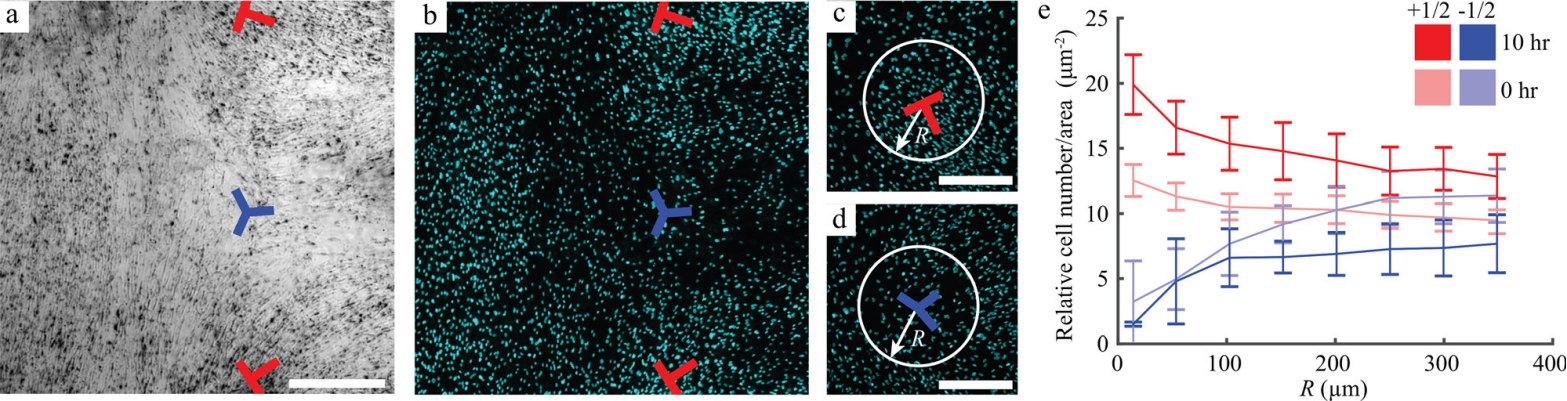
Mesothelial cell density at the topological defects. (a) and (b) Phase contrast image (a) and DAPI (4′,6-diamidino-2-phenylindole) stain (b) of a −1/2 defect adjacent to two +1/2 defects. Qualitatively, cell density is higher at the +1/2 defects and lower at the −1/2 defect. (c) and (d) Representative images of a +1/2 and −1/2 defects with a circle of radius *R* drawn around them. (e) Cell density was quantified for different circles of radius *R* for both types of defects at 0 h and 10 h. The graphs show mean ± standard deviation for *n *=* *6 + 1/2 defects and *n *=* *3 − 1/2 defects. Scale bars: 500 *μ*m.

The velocity data show a net inward flow at +1/2 defects and a net outward flow at −1/2 defects. Such flow patterns would be expected to change the local cell density with an increase and decrease in cell density expected at +1/2 and −1/2 defects, respectively. Consistent with this reasoning, we often observed greater cell density at +1/2 defects and lower density at −1/2 defects [Figs. 3(a) and 3(b), Videos 1 and 2]. To quantify this observation, we measured the average cell density at two time points separated by 10 h. The data were analyzed by quantifying the cell density within circles of radius *R* centered on each defect [Figs. 3(c) and 3(d)] and varying *R* from 10 to 350 *μ*m [Fig. 3(e)]. Slopes of the graph of cell density vs *R* were quantified by linear regression. For +1/2 defects, the slopes and 95% confidence intervals were −0.005 [−0.014, 0.004] at 0 h and −0.018 [−0.029, −0.006] at 10 h. The negative confidence intervals at the 10 h time point indicate greater density at the center of the +1/2 defects. A comparison of cell density nearest to the +1/2 defects (i.e., corresponding to the smallest value of *R*) showed that the data at 0 and 10 h were statistically different (*p *=* *0.004, rank sum test), indicating the accumulation of cells occurred during the experimental timeframe at +1/2 defects, consistent with the inward cell velocity at this type of defect observed in Fig. 2. For −1/2 defects, slopes and confidence intervals were 0.031 [0.023, 0.038] and 0.014 [0.006, 0.022] at the 0 and 10 h time points, respectively. The positive slopes for both the time points indicate that cell density was lower at the center of the −1/2 defects.

### Mesothelial clearance near topological defects

**FIG. 4.**
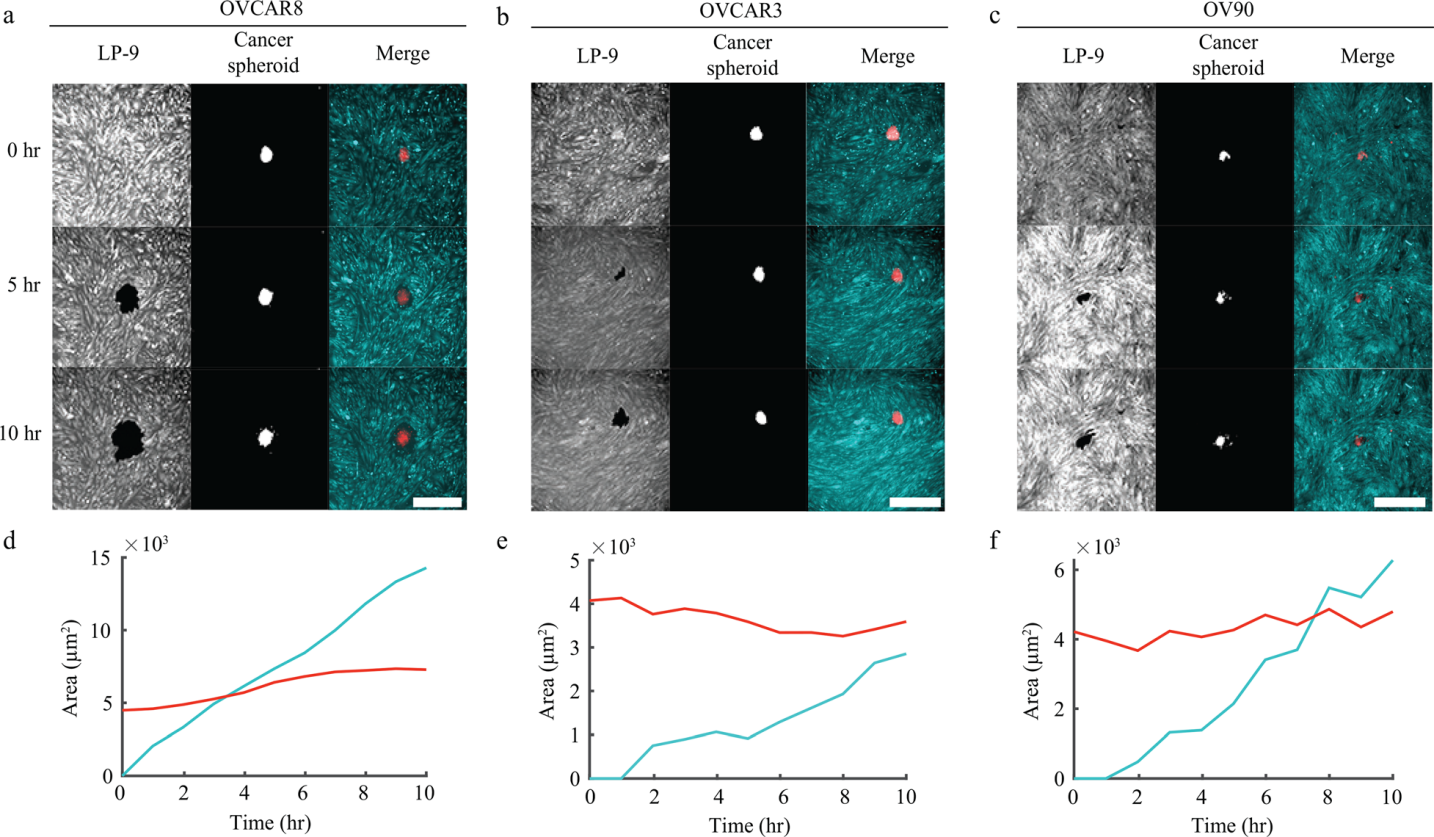
Clearance by various ovarian cancer cell lines on defect-free locations in the mesothelial layer. (a)–(c) Representative images showing clearance of mesothelial cells by OVCAR8 (a), OVCAR3 (b), and OV90 (c) cells. (d)–(f) Corresponding area cleared in the mesothelial cells (blue) and area of the cancer cell spheroids (red) over time for OVCAR8 (d), OVCAR3 (e), and OV90 (f) cells. Scale bars: 500 *μ*m.

In layers of a single cell type, the defect-induced changes in cell density can cause cells to extrude from the layer or holes to form in the cell layer.^16,17,19^ These observations combined with our data showing defect-induced changes in cell density in the LP-9 layer led us to hypothesize that topological defects would affect the rate at which ovarian cancer cells clear the LP-9 layer. We chose three ovarian cancer cell lines, OVCAR8, OVCAR3, and OV90, and used an experimental model of mesothelial clearance.^25^ As ovarian cancer cells metastasize as both single cells and aggregates of cells, we chose to generate spheroids of cancer cells, which were labeled with CellTracker Deep Red and seeded upon confluent layers of LP-9 cells that had been labeled with CellTracker Blue. Time lapse fluorescence microscopy of different colors enabled the LP-9 cells and cancer cell spheroids to be imaged independently. The imaging revealed cancer cell invasion into the mesothelial layer occurring over a period of several hours. During invasion, the LP-9 cells were cleared away, resulting in free space that was filled by the spreading cancer cells [Figs. 4(a)–4(c)]. Areas of both the spheroid and the cleared space were measured. The spheroid size remained relatively constant over time, while the cleared area increased approximately linearly over time [Figs. 4(d)–4(f)]. The rate of clearance was computed by fitting the cleared area over time to a line and determining the slope. To account for spheroids of different sizes (and for the fact that larger spheroids cleared larger areas of mesothelial cells), the rate of clearance was normalized by the initial size of the spheroid, giving a normalized clearance rate.

**FIG. 5.**
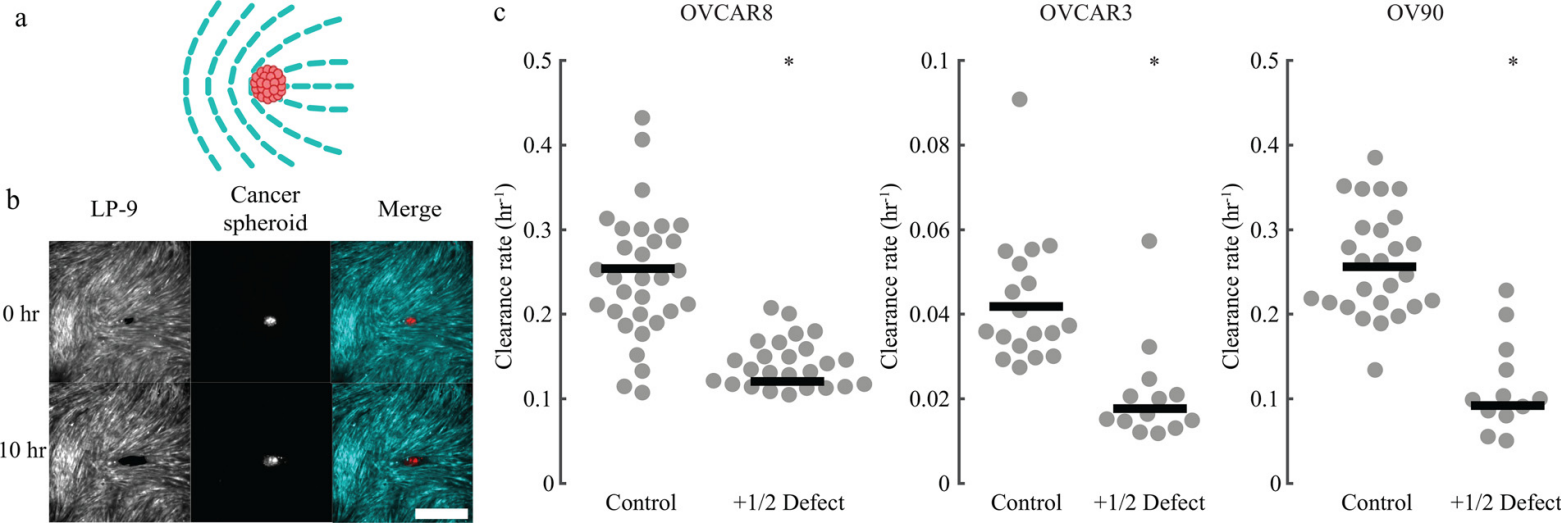
Mesothelial clearance near +1/2 defects. (a) Cartoon showing a cancer cell spheroid near a +1/2 defect. (b) Representative images of clearance of an OVCAR8 cell spheroid at a +1/2 defect in the mesothelial layer. (c) Normalized clearance rate at regions having +1/2 defects compared to defect-free control regions for OVCAR8 (*p *<* *0.001), OVCAR3 (*p *<* *0.001), and OV90 (*p *<* *0.001) spheroids. Each dot represents an independent spheroid. Lines show medians. Scale bar: 500 *μ*m.

The median normalized clearance rates for many spheroids at control locations without topological defects were 0.25, 0.041, and 0.28 h^−1^ for OVCAR8, OVCAR3, and OV90 cells, respectively (Fig. 5). We then seeded ovarian cancer spheroids on top of +1/2 defects and measured the normalized clearance rates [Figs. 5(a) and 5(b)]. The normalized clearance rates on defects were smaller than clearance rates on control locations having no topological defects [Fig. 5(c)]. This finding did not depend on our choice to normalize the data, as the rate of clearance was smaller for non-normalized data as well [Figs. 12(a)–12(c)], consistent with the fact that the average spheroid size remained the same at different locations [Figs. 12(d)–12(f)]. Intriguingly, the observation of a reduced rate of clearance on +1/2 defects was consistent across all three cell types. This finding combined with the observation of inward flow [Figs. 2(b) and 2(c)] and increased local density [Fig. 3(e)] of mesothelial cells at +1/2 defects indicates the importance of physical factors (cell velocity and density) in mesothelial clearance.

**FIG. 6.**
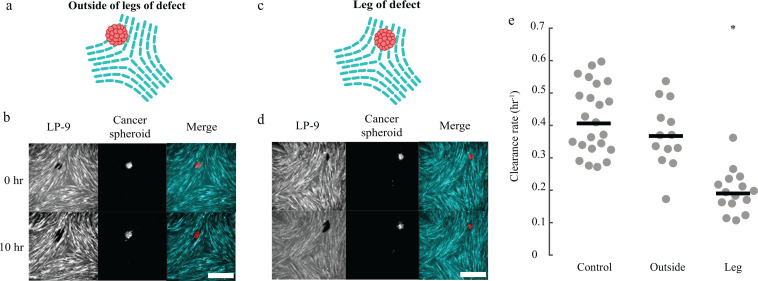
Mesothelial clearance near −1/2 defects. (a) Cartoon showing a cell spheroid outside of the legs of a −1/2 defect. (b) Representative images of clearance of an OVCAR8 cell spheroid outside the legs of a −1/2 defect. (c) Cartoon showing a cell spheroid on a leg of a −1/2 defect. (d) Representative images of clearance of an OVCAR8 cell spheroid on the leg of a −1/2 defect. (e) Normalized clearance rate of OVCAR8 spheroids at defect-free control regions and locations outside and on the legs of −1/2 defects. Each dot represents an independent OVCAR8 spheroid. Lines show medians. The clearance rate on the legs was statistically different from both control and outside regions (*p *<* *0.001). Scale bar: 500 *μ*m.

**FIG. 7.**
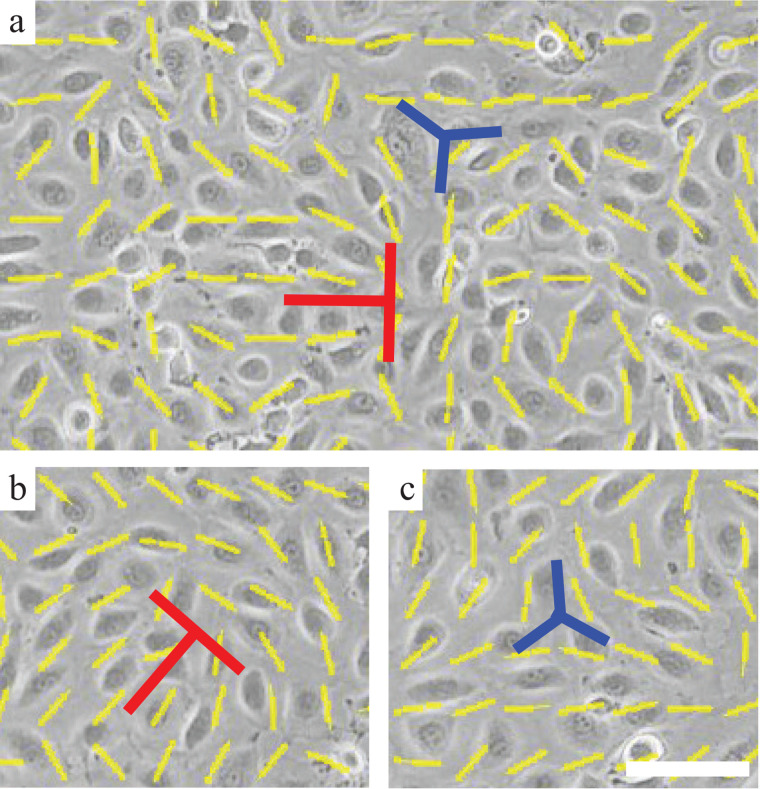
Topological defects in monolayers of primary human mesothelial cells. (a) Image of the primary human mesothelial cells showing a +1/2 and a −1/2 defect. The yellow lines indicate cell orientation. (b) Image of primary human mesothelial cells showing a +1/2 defect. (c) Image of primary human mesothelial cells showing a −1/2 defect. Cells were isolated from a benign omentum obtained from autopsy from an 88-year-old Caucasian female using methods described previously.^27^ Scale bar: 100 *μ*m.

**FIG. 8.**
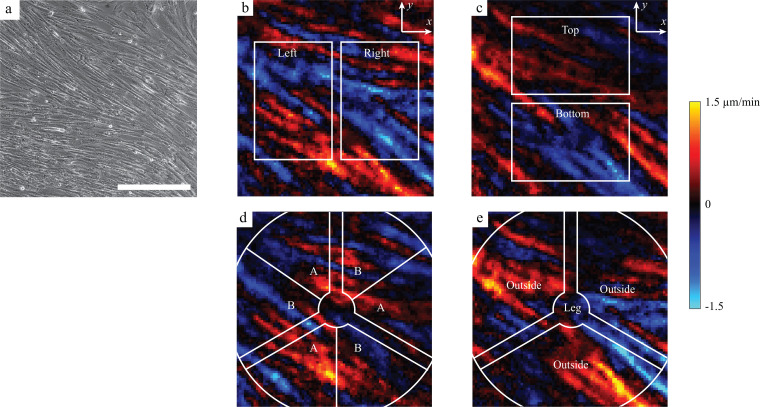
Collective mesothelial cell migration patterns at defect-free control regions. (a) Representative image of a defect-free control region in the LP-9 cell layer. (b) Colormap of the *x* component of cell velocity averaged over 24 h. (c) Colormap of the *y* component of cell velocity averaged over 24 h. Velocities were averaged within 500 × 750 *μ*m^2^ regions as identified by the white boxes. (d) Colormap of the angular component of cell velocity averaged over 24 h. Velocities were averaged within the six regions on either side of the three legs of the −1/2 defect. Data points within a distance of 250 *μ*m from the center of the defect or within 45 *μ*m from the legs of the defect were excluded. (e) Colormap of the radial component of cell velocity averaged over 24 h. Velocities were averaged within the four regions shown in panel (e). The resulting data for all control regions are shown in Figs. 2 and 10. Scale bar: 500 *μ*m.

**FIG. 9.**
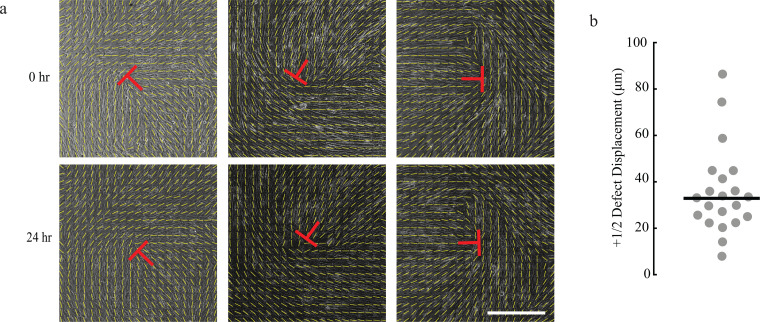
Motion of +1/2 defects in the LP-9 cell layer. (a) Three representative phase contrast images of LP-9 cells with +1/2 defects with cell orientations overlaid at 0 and 24 h. Scale bar: 500 *μ*m. (b) Total displacement of +1/2 defects (*n *=* *21) measured by taking the distance between the positions of the defects at 0 and 24 h.

**FIG. 10.**
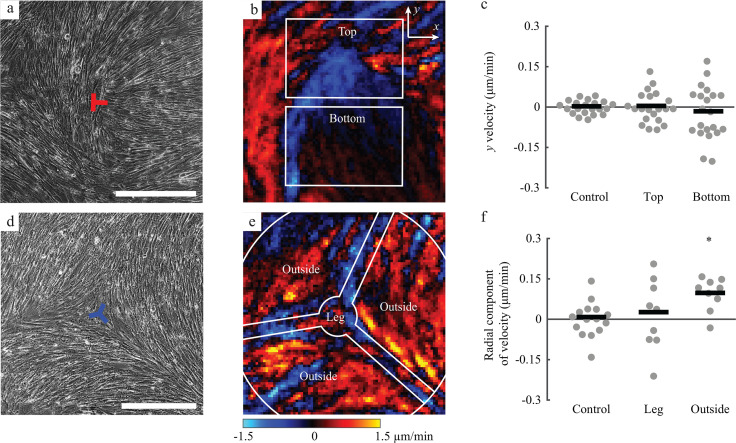
Collective cell migration patterns near defects in the mesothelial cell layer. (a) Representative image of a +1/2 defect. (b) Colormap of the *y* component of cell velocity near the +1/2 defect shown in panel (a) averaged over 24 h. (c) Velocities were averaged within regions of width 750 *μ*m and height 500 *μ*m on the top and bottom of the defect, as identified by the white boxes in panel (b). The plot shows the average *y* velocity of control positions having no defect (*n *=* *21) and at the top and bottom sides of +1/2 defects (*n *=* *21, *p *>* *0.05). (d) Representative image of a −1/2 defect. (e) Colormap of the radial component of cell velocity near the −1/2 defect shown in panel (d) averaged over 24 h. (f) Radial velocities were averaged on the legs and outside the legs, as shown in panel (e). The plot shows the average radial velocity of control positions (*n *=* *15) and of positions on the leg (*p *>* *0.05) and outside the leg (*p *<* *0.05) of −1/2 defects (*n *=* *9). Scale bar: 500 *μ*m.

At −1/2 defects, the analysis of the velocity fields showed cells moving toward the legs of each defect [Figs. 2(e) and 2(f)]. Hence, we performed a refined analysis of clearance at −1/2 defects by quantifying separately the clearance rates of spheroids located outside the legs of a −1/2 defect [Figs. 6(a) and 6(b)] and on the legs [Figs. 6(c) and 2(d)]. Compared to control, clearance rates of spheroids outside the legs were no different, but for spheroids located on the legs, the clearance rate was smaller by a factor of ∼2 [Figs. 6(e), 12(g), and 12(h)]. Hence, both +1/2 and −1/2 topological defects within the LP-9 cell layer affected the rate of mesothelial clearance.

## DISCUSSION

In contrast to most solid tumors, ovarian cancer primarily metastasizes by transport through the peritoneal fluid to colonize new metastatic sites. Understanding the mechanisms that support and resist this process may identify new approaches to slow or stop metastatic spread in patients. Numerous *in vitro* studies have demonstrated that mesothelial cells serve as a likely barrier to peritoneal metastasis, as more tumor cells attach to extracellular matrix than to mesothelial cells^26^ and mesothelial cells slow invasion in a transwell assay.^27^ Prior work has identified some of the molecular components that a tumor cell can utilize to attach to this barrier, including vitronectin,^28^ hyaluronic acid,^3^ mesothelin,^29^ fibronectin,^27,30^ and P-selectin.^4^ However, following attachment, the tumor cell must clear through the mesothelial cell layer—if the tumor cells are unable to embed into the peritoneal tissue, the ability to set up a new metastatic niche will be interrupted.

Here, we provide evidence for a biophysical regulator of mesothelial clearance with our observation of converging, inward cell velocities at the center of +1/2 defects and on the legs of −1/2 defects in mesothelial cell layers. At these locations of converging flow, the rate of clearance by cancer cells was reduced. Prior studies demonstrated roles for biophysical mechanisms in the process of mesothelial clearance. First, attachment and clearance of individual tumor cells occurred preferentially at mesothelial cell–cell junctions.^26,28^ Unlike our analysis of mesothelial cell topography, these two studies did not determine if cells preferentially cleared at specific cell–cell alignments (e.g., between the long axis of two neighboring cells). Second, tumor cell invasion was only slowed by the presence of a confluent monolayer of mesothelial cells; treatment with mesothelial cell-conditioned media did not have the same effect.^27^ Third, the integrin-dependent activation of myosin has been shown to be essential for mesothelial clearance.^11^

Given the heterogeneity that ovarian cancer is known for, most prior studies and our own examined multiple tumor cell lines in parallel. OVCAR3 was reported to be able to clear the mesothelial cell barrier,^5^ but to our knowledge, OVCAR8 and OV90 have not been previously examined. We selected these cell lines as they have been classified as genomically consistent with patient tumors.^31^ OVCAR3 had a baseline clearance rate on control positions of the cell layer that was approximately five times smaller than OVCAR8 or OV90 cells. Prior comparisons across tumor cell lines have of course demonstrated relationships between cell behaviors and the levels of key proteins in the mechanism of interest; for example, the level of epidermal growth factor receptor (EGFR) ligands predicted sensitivity to anti-EGFR therapies,^32^ and receptor levels predicted sensitivity to macrophage-secreted factors.^33^ A previous study identified a relationship between the expression of mesenchymal genes in tumor cells (e.g., *SNAI1*, *TWIST1*, and *ZEB1*) and the extent of clearance.^5^ However, nearly all ovarian cancer cell lines studied have the ability to clear the mesothelial layer to some extent, and prior studies have not examined the variability of clearance with respect to proximity to different mesothelial topologies. Our results demonstrated that clearance on the +1/2 defects was consistent across the three different ovarian cancer cell lines, suggesting that some of the biochemical variations between cell lines converge into shared biophysical mechanisms.

This observation suggests that topics from physics may be informative for mesothelial clearance. Physics-based theoretical models originally developed for active liquid crystals and subsequently applied to bacteria, fibroblasts, and epithelial cells have related the orientation field of the cells to the velocity field.^16,17,19,21^ In our experiments, the cell velocities shared some similarities with theoretical predictions. For +1/2 defects, cells along the comet tail migrated toward the center of the defect. For −1/2 defects, cells outside the legs moved radially outward. A difference with the theory of active matter is that the theory predicts that the inward and outward flow at each defect is balanced such that the net flow is zero. In contrast, the experimental data revealed net inward and outward flows at the center of +1/2 and −1/2 defects, which caused the local increase and decrease in cell density, respectively. Similar inward/outward cell velocities and local changes in density at topological defects have been observed in other studies, though the precise details of the velocity fields have differed. For example, although a net outward cell velocity at −1/2 defects has been commonly observed,^16,18,19^ some studies identified a net inward cell velocity at +1/2 defects,^16,19^ whereas another identified a net outward cell velocity.^18^ Hence, cell layers do not exactly match the standard theory from the field of active liquid crystals.

One difference may be the presence of an anisotropic resistance to motion (referred to as “friction” in the theoretical models) caused by the elongated cell shapes.^16,19^ The friction is thought to be greater for motion perpendicular to the axis of the cell as compared to motion along the axis, and, because cell orientations change abruptly at the defects, the differential friction breaks the symmetry in the velocity fields, thereby causing net inward or outward flows at +1/2 and −1/2 defects, respectively. Experiments have not yet confirmed that friction depends on the orientation of the cell, leaving open the possibility that some other mechanism is responsible for the net inward and outward cell flows near the defects. Moreover, friction alone does not describe all of our observations. For example, on the left side of a +1/2 defect, the theory with anisotropic friction predicts reduced velocity, whereas in our experiments, the velocity was not only reduced but also reversed its direction. The theory, therefore, is a useful starting point but does not fully capture the migration of LP-9 cells. More important than these differences between theory and experiments is the physical picture that emerges from quantifying cell velocities and local cell densities—topological defects cause a net inward or outward cell velocity field that creates a local increase or decrease in cell density, in turn affecting cell extrusion,^16,19^ causing formation of holes,^19^ and, in our data, impacting mesothelial clearance.

In summary, this work presents a new biological application of topological defects in cell layers: the cell velocity field defined by defects affects the rate of mesothelial clearance by ovarian cancer cells. In this study and others, concepts from topology and active matter offer important new perspectives on biological research. As the interplay between physical cell properties—orientation, velocity, force—and tissue function is complicated, the field of soft matter physics provides numerous opportunities to discover new connections between physics and tissue function.

## METHODS

### Cell culture

The OVCAR3 and OV-90 lines were purchased from ATCC (Manassas, VA, USA), and OVCAR8 were obtained from the NCI 60 panel (NIH, Bethesda, MD). All cancer cell lines were cultured in a 1:1 ratio of Medium 199 (ThermoFisher Scientific, Waltham, MA) and MCDB 105 (MilliporeSigma, St. Louis, MO) supplemented with 10% fetal bovine serum (Corning, Inc., Corning, NY). LP-9 mesothelial cells were obtained from Coriell and maintained in a 1:1 ratio of Medium 199 and Ham's F-12 (Corning) supplemented with 15% fetal bovine serum (Corning), 10 ng/ml epidermal growth factor (MilliporeSigma), and 0.4 *μ*g/ml hydrocortisone (MilliporeSigma). All cells were grown at 37 °C in humidified 5% CO_2_. Cells were confirmed to be negative for mycoplasma before freezing into separate lots, and each lot was used for up to 22 passages.

### Microscopy

Microscopy was performed on an Eclipse Ti microscope with a 10× numerical aperture 0.5 objective and a 4× numerical aperture 0.13 objective (Nikon Instruments, Melville, NY) in phase contrast and fluorescence modes. Images were captured with an Orca Flash 4.0 digital camera (Hamamatsu, Bridgewater, NJ) using NIS-Elements Ar software (Nikon). Time-lapse imaging was performed in a custom-built cage incubator that maintained the cells in a humid 37 °C, 5% CO_2_ environment.

### Live cell imaging for cell velocity analysis

To quantify cell velocities, LP-9 cells (0.5 × 10^6^) were plated onto collagen I (0.1 mg/ml) and fibronectin (0.5 *μ*g/ml)-coated plastic dishes five days before the experiment. The use of collagen I and fibronectin was motivated by immunofluorescent staining showing the presence of these proteins in the mesothelial layer of benign human omentum (Fig. 13), and prior dot blots^34^ that indicated a ratio of fibronectin to collagen I of 0.25:100 to 0.5:100. Phase contrast images were captured every 10 min for 24 h, and cell velocities were computed by applying Fast Iterative Digital Image Correlation.^35^ Consecutive images were correlated, and the resulting displacements were divided by time to compute velocity. Subsets of 64 × 64 pixels were used with a spacing of 16 pixels (10 *μ*m).

For each +1/2 defect, the image and velocity field were rotated such that the comet tail pointed to the right. Rectangular boxes of size 750 × 500 *μ*m were drawn immediately to the left, right, top, and bottom of the defect. Averages of the *x* and *y* components of velocities were subsequently computed in the rectangular boxes. Rectangular boxes of the same size were used to analyze control datasets as well.

For each −1/2 defect, the radial and angular components of velocity were computed. To analyze the angular component, the image was separated into six regions [labeled as A and B in Fig. 2(e)]; data within the central 250 *μ*m and within 45 *μ*m of the center of each leg were excluded; all other data points within 1800 *μ*m of the center of the defect were included. To analyze the radial velocity, the image was separated into two regions, on the legs (defined as being within 45 *μ*m from the center of a leg) and outside the legs.

**FIG. 11.**
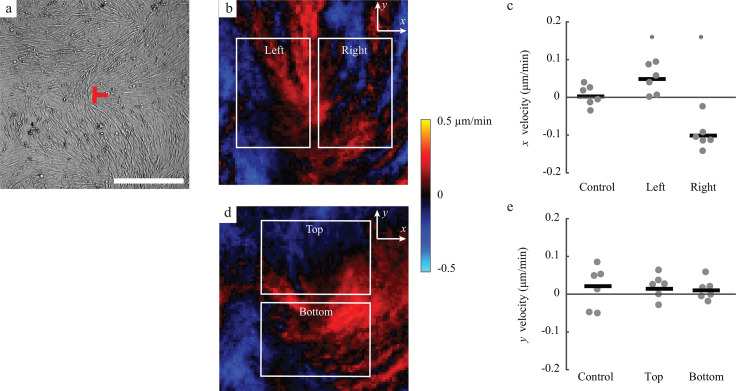
Collective cell migration patterns near +1/2 defects in the mesothelial cell layer on 3 kPa polyacrylamide substrates. (a) Representative image of a +1/2 defect in an LP-9 monolayer on a 3 kPa polyacrylamide substrate. (b) Colormap of the *x* component of cell velocity near the +1/2 defect shown in panel (a) averaged over 24 h. (c) Velocities were averaged within regions of width 500 *μ*m and height 750 *μ*m on the left and right of the defect as identified by the white boxes in panel (b). The plot shows the average *x* velocity of control positions having no defect (*n *=* *6) and in regions to the left (*p *<* *0.05) and right (*p *<* *0.05) of +1/2 defects (*n *=* *6). (d) Colormap of the *y* component of cell velocity near the +1/2 defect shown in panel (a) averaged over 24 h. (e) Velocities were averaged within regions of width 750 *μ*m and height 500 *μ*m on the top and bottom of the defect as identified by the white boxes in panel (d). The plot shows the average *y* velocity of control positions having no defect (*n *=* *6) and of positions on the top and bottom sides of +1/2 defects (*n *=* *6, *p *>* *0.05). Scale bar: 500 *μ*m.

**FIG. 12.**
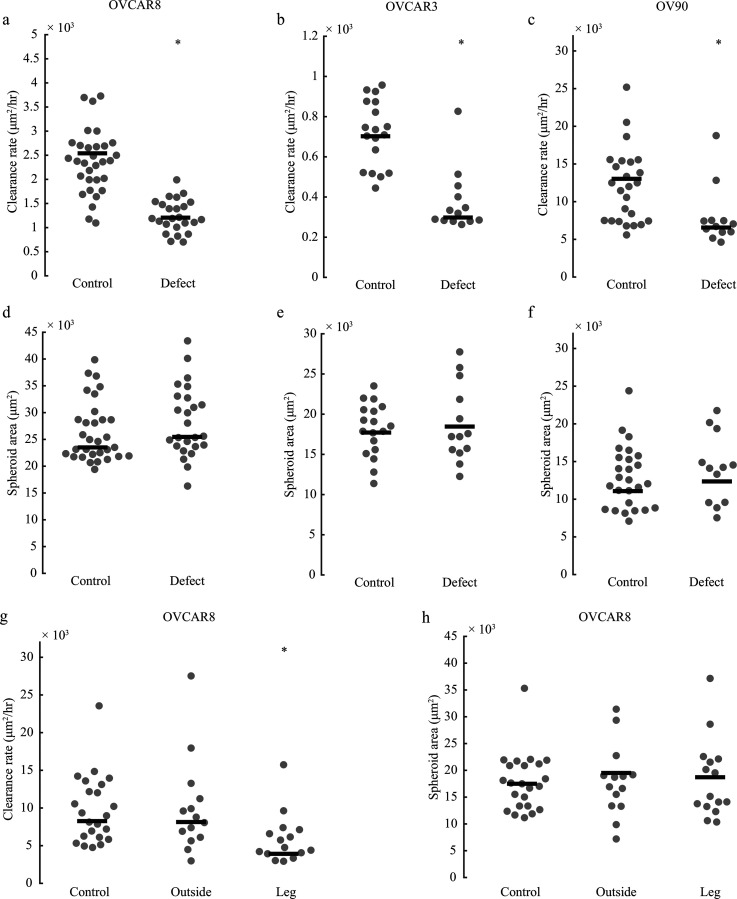
Non-normalized mesothelial clearance data. (a)–(c) Non-normalized clearance rate by the three types of cancer cell spheroids at defect-free control regions or regions having +1/2 defects. Each dot represents an independent ovarian cancer spheroid. Lines show medians. For all types of cancer cell spheroids, non-normalized clearance rates on +1/2 defects were statistically different from control (*p *<* *0.001). (d)–(f) Initial spread areas of the three types of cancer cell spheroids at defect-free control regions or regions having +1/2 defects. (g) and (h) Non-normalized clearance rate (g) and spheroid area (h) on outside and leg regions of −1/2 defects. The clearance rate on leg regions is statistically different from control and regions outside the legs (*p *<* *0.05). Each dot represents an independent ovarian cancer spheroid. Lines show medians.

**FIG. 13.**
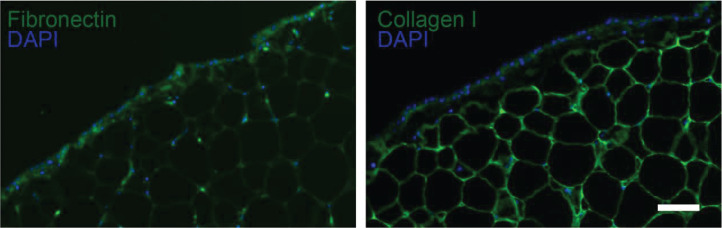
Immunofluorescent staining of benign human omentum obtained from archived pathology samples through a protocol approved by the UW-Madison IRB. The mesothelial layer is the thin layer of cells covering the larger, circular adipocytes. This region stained positive for both fibronectin and collagen type I. Dot blots of benign human omentum^34^ indicated a ratio of fibronectin to collagen I in this region of 0.25:100 to 0.5:100. Scale bar: 100 *μ*m.

For experiments in Fig. 11, polyacrylamide gels were prepared with Young's modulus of 3 kPa and thickness of 75 *μ*m. A gel solution of 5.5% weight/volume (w/v) acrylamide (Biorad Laboratories, Hercules, CA) and 0.2% w/v bisacrylamide (Biorad) was prepared, and 20 *μ*L was pipetted onto No. 1.5 thickness glass-bottom dishes (Cellvis, Mountain View, CA). A glass coverslip (18 mm diameter circle) was placed on each gel and removed after the gel solution was polymerized. The top surface of the gel was coated with collagen I (0.1 mg/ml) and fibronectin (0.5 *μ*g/ml) using the covalent cross-linker sulfo-SANPAH (ProteoChem, Hurricane, UT). To quantify cell velocities, LP-9 cells (0.5 × 10^6^) were seeded onto coated polyacrylamide gels five days before the imaging. Phase contrast images of +1/2 defects were captured every 10 min for 10 h. Velocities were calculated and analyzed in the same way as for data collected on plastic dishes.

### Cell density analysis

LP-9 cells were seeded on 6-well plates coated with collagen I (0.1 mg/ml) and fibronectin (0.5 *μ*g/ml). Samples were fixed 5 days after seeding using 4% paraformaldehyde in the phosphate buffered saline (PBS) at room temperature for 10 min followed by 10 min of permeabilization in PBS containing 0.2% Triton X-100. Specimens were washed for 5 min three times in PBS, and in the last wash, 600 nM DAPI (4′,6-diamidino-2-phenylindole) was added. To analyze the images for cell densities, 15 nuclei from each dish were randomly selected, and their fluorescent intensities were averaged to compute the average fluorescent intensity for a single cell. The radius-dependent average cell density was calculated by summing the fluorescent intensity within a circle of radius *R* and dividing by the averaged single-cell fluorescent intensity.

### Live cell imaging for spheroid-induced mesothelial clearance assay

A silicone mold of an AggreWell 400 24-well plate (Stemcell Technologies, Vancouver, Canada) was created, and 1.5% agarose was cast onto the silicone mold. Upon curing, the agarose replicated the surface of the AggreWell plate and was used for making spheroids. Ovarian cancer cells were stained with 10 *μ*M CellTracker Deep Red for 30 min (ThermoFisher), and 4.8 × 10^5^ cells were added to each well. The plates were centrifuged (10 min at 100*g*) and left in the incubator for 48 h to form spheroids. Upon collection, each well was gently washed with serum free cancer cell medium and single cells were removed by filtering with a 40 *μ*m strainer. The final spheroid concentration was estimated by a hemocytometer.

The LP-9 cells (0.5 × 10^6^) were plated on 6-well plastic bottom dishes coated with collagen I (0.1 mg/ml) and fibronectin (0.5 *μ*g/ml). Cells were maintained in the culture until they became aligned with their neighbors (typically 3–4 days after plating). When cell alignment was observed, the cell medium was switched to imaging medium (containing a 1:1 ratio of Medium 199 and MDCB 105 supplemented with 1% fetal bovine serum) for an additional 24 h before imaging. On the day of experiment, LP-9 cells were stained with 10 *μ*M CellTracker Blue for 30 min (ThermoFisher).

In the mesothelial clearance assay, approximately 100 spheroids were added to a confluent LP-9 layer, allowed to attach for 40 min, and washed with imaging medium (1:1 Medium 199:MDCB 105 supplemented with 1% fetal bovine serum) to remove unattached spheroids. The attached spheroids were imaged every hour for 15 h by phase contrast and fluorescence microscopy in the imaging medium. For +1/2 defects, any spheroid located within 750 *μ*m of the defect and on the side of the comet tail was imaged. For −1/2 defects, any spheroid located within 750 *μ*m of the center of the defect was imaged.

To quantify spread areas of the spheroids, fluorescent images of the spheroids were binarized using ImageJ to extract the spheroid spread areas during imaging. The cleared area within the LP-9 layer was measured manually in ImageJ.

### Statistical analysis

In the velocity analysis, each dot represents an independent defect position, and statistical comparisons were performed using one-way analysis of variance with Bonferroni correction for multiple comparisons. To analyze cell densities, slopes were computed by linear regression, and comparisons at individual time points were made using a Wilcoxon rank sum test. In the mesothelial clearance assay, each dot represents an independent cancer spheroid, and statistical comparisons were performed using the Wilcoxon rank sum test or, for multiple comparisons, the Kruskal–Wallis test with Bonferroni, because not all datasets were normally distributed based on the Anderson–Darling test. The symbol * is used in the figures to indicate statistical differences in comparison to control, and the reported *p* values in the figure legends are in comparison to control unless stated otherwise.

## SUPPLEMENTARY MATERIAL

See the supplementary material for Videos 1 and 2 and their legends.

## Data Availability

The data that support the findings of this study are available from the corresponding authors upon reasonable request.

## APPENDIX: SUPPLEMENTAL FIGURES

Figures 7–13 appear below.
